# Efficient and precise generation of Tay–Sachs disease model in rabbit by prime editing system

**DOI:** 10.1038/s41421-021-00276-z

**Published:** 2021-07-06

**Authors:** Yuqiang Qian, Ding Zhao, Tingting Sui, Mao Chen, Zhiquan Liu, Hongmei Liu, Tao Zhang, Siyu Chen, Liangxue Lai, Zhanjun Li

**Affiliations:** 1 Key Laboratory of Zoonosis Research, Ministry of Education, College of Animal Science, Jilin University, Changchun, Jilin, China; 2 CAS Key Laboratory of Regenerative Biology, Guangdong Provincial Key Laboratory of Stem Cell and Regenerative Medicine, South China Institute for Stem Cell Biology and Regenerative Medicine, Guangzhou Institutes of Biomedicine and Health, Chinese Academy of Sciences, Guangzhou, Guang Dong, China

**Keywords:** Biological techniques, Metabolomics

Dear Editor,

Tay–Sachs disease (TSD) is a progressive neurodegenerative disorder due to an autosomal recessively inherited deficiency of β-hexosaminidase A (HexA)^1^. The four-bases (TATC) insertion in exon 11 of the *HEXA* (*HEXA* ins TATC) accounts for 80% of Tay–Sachs disease from the Ashkenazi Jewish population^2^. However, no typical clinical phenotypes, such as neurological abnormalities, the restricted pattern of distribution of GM2-ganglioside and membranous cytoplasmic bodies in the brain, were observed in *HEXA*^−/−^ mouse models, due to the difference in the ganglioside degradation pathways in mice and human^3^. Thus, it is desired to generate an ideal animal model to accurately mimic *HEXA* ins TATC in TSD patients. CRISPR–Cas9 system-mediated HDR^4^ has been used to generate the mutation of *HEXA* ins TATC, however, low efficiency and high indels impede its application.

Recently Anzalone et al.^5^ described a “search-and-replace” genome editing technology named prime editing (PE) that mediates 12 possible base-to-base conversions, without requiring DSBs or donor DNA templates in human cells. In addition, a previous study showed that, compared to mice, the late onset of TSD in adult rabbits^6^ shared more similarities with human regarding physiology, anatomy, and genetics^7^. Thus, we generated a novel TSD rabbit model using the PE system, and characterized the typical phenotype of muscle weakness, ataxia, and mental disorders in the *HEXA* ins TATC rabbit model.

We first validated the editing efficiencies of PEs (PE2, PE3, PE3b) in HEK293FT cells at fifteen loci: five loci for base insertion, eight loci for base substitutions, and two loci for base deletion (Supplementary Table S1). Sanger sequencing results showed that the base insertion at a frequency from 4% to 22% (Fig. 1a and Supplementary Fig. S2), the base substitutions at a frequency from 4% to 36%, and the base deletion at a frequency from 7% to 12% were determined using PEs (Supplementary Figs. S1 and S2), respectively. These results indicate that PEs were effective in generating base insertion, substitution, and deletion in HEK293FT cells.

**Fig. 1:**
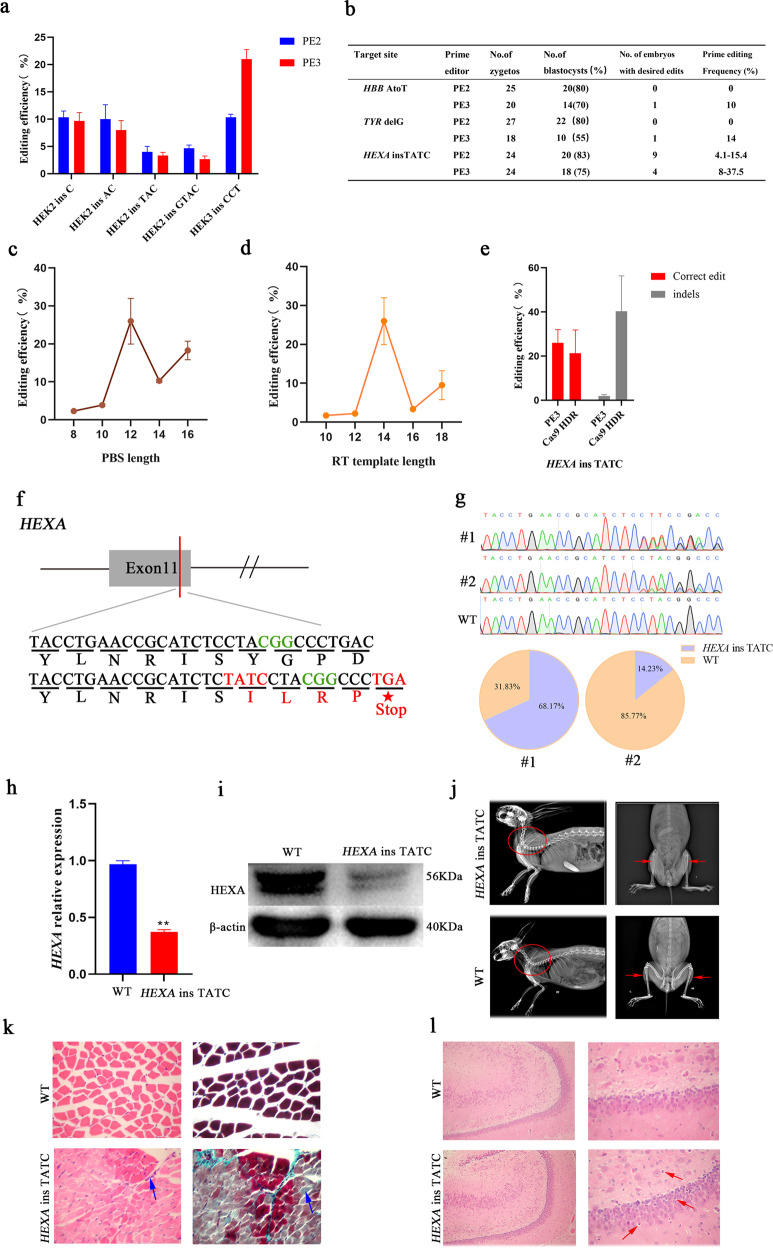
PE induced efficient and precise gene editing in rabbit. **a** The base insertion efficiency of PE system in HEK293FT cells. **b** PE induced efficient and precise gene editing in rabbit embryos. **c** Editing frequency (*HEXA* ins TATC) of PegRNA screening with PBS length (8–16 nt) in rabbit embryos. **d** Editing frequency (*HEXA* ins TATC) of PegRNA screening with RT template length (10–18 nt) in rabbit embryos. **e** Editing frequency (*HEXA* ins TATC) of CRISPR–Cas9 system-mediated HDR compare with PE3. **f** The target sequence at *HEXA* locus by PE system. The PAM and sgRNA target sequences are shown in green and black, target mutation (red), frameshift mutation leads to PTC mutation (red and red star). **g** Editing frequency determination of *HEXA* ins TATC rabbit by deep sequencing. **h** Expression of *HEXA* gene was determined by qRT-PCR. **i** HEXA protein was determined by western blot. **j** X-ray radiography of WT and *HEXA* ins TATC rabbits. Red circle, increased cervical lordosis; Red arrows, clasping of the limbs. **k** Masson’s trichrome staining of gastrocnemius from WT and *HEXA* ins TATC rabbits. Blue arrow highlights the myopathy with fibrosis and inflammatory cell infiltration. **l** HE staining of hippocampus from WT and *HEXA* heterozygous rabbits. The red arrow highlights the enlargement of perineural space.

Next, we tested the efficiency of the PE system in rabbit embryos at three gene loci of *HEXA*, *HBB*, and *TYR*, which are associated with clinical diseases in ClinVar data^8^ (Supplementary Table S2). Sanger sequencing results showed that 9 of 20 desired *HEXA* ins TATC were determined using PE2 with the efficiency of 4.1%–15.4%, while the efficiency is 8%–37.5% using PE3. In addition, 1 of 14 desired *HBB* with an efficiency of 10% and 1 of 10 desired *TYR* with an efficiency of 14% were generated using PE3, while there is no desired mutation was detected for these two sites using PE2 (Fig. 1b and Supplementary Fig. S3).

We then targeted the *HEXA* ins TATC to test the efficiency of the PegRNA PBS length (8–16 nt) and RT template length (10–18 nt) in rabbit embryos. TIDE analyzing^9^ revealed significantly higher editing efficiencies by using PegRNA with 12 nt PBS and 14 nt RT template (Fig. 1c, d and Supplementary Table S3). Additionally, the significantly increased undesired indels were determined by using CRISPR–Cas9 system-mediated HDR (Fig. 1e and Supplementary Tables S3, S8), which is consistent with the previous study^4^. Thus, PE3 with 12 nt PBS and 14 nt RT template was used for the generation of *HEXA* ins TATC rabbits in the following study.

The *HEXA* ins TATC introduces a premature termination codon (PTC) in exon 11, which leads to deficient activity of the hexosaminidase A (HexA)^10^ (Fig. 1f). In this study, 2 of 4 *HEXA* ins TATC rabbits were determined using Sanger sequencing and targeted deep sequencing, with the 68.17% and 14.23% mutation efficiency for #1 and #2 pups, respectively (Fig. 1g). Furthermore, no sgRNA sequence-depended off-target mutations in *HEXA* ins TATC rabbits were found by deep sequencing (Supplementary Fig. S4a, b), suggesting the accuracy of PE system-mediated *HEXA* ins TATC mutations in rabbits.

Furthermore, the heritability of *HEXA* ins TATC in rabbits was determined by Sanger sequencing (Supplementary Fig. S5), qRT-PCR (Fig. 1h), and western blot (Fig. 1i). The results showed a significantly reduced HEXA in *HEXA* ins TATC rabbits compared with WT controls. The typical phenotypes of the increasingly frequent of head raising, convulsions (Supplementary Fig. S6a and Movies S1, S2), abnormal gait with decreased sway length (Supplementary Fig. S6b and Movies S1, S2), clasping of the limbs, and increased cervical lordosis (Fig. 1j), muscle fibrosis (Fig. 1k) and enlargement of perineural space (Fig. 1l) were also determined in *HEXA* ins TATC rabbits when compared with WT controls. These phenotypes were similar with late-onset or chronic adult gangliosiderosis in TSD patient exhibiting as limb-girdle weakness, followed by the development of ataxia and progressive neuromuscular weakness^11^.

In summary, this study for the first time verified the feasibility of PE system-mediated base insertions, deletions, and conversions in rabbit. This ideal and novel HEXA ins TATC rabbit model would be beneficial for the pathogenic mechanism study and drug screening to treat TSD in the future.

## Supplementary information

supplymentary data

Supplementary Movie S1

Supplementary Movie S2
