# Supplementary material for: Efficient and precise generation of Tay–Sachs disease model in rabbit by prime editing system
Source: Cell Discov. 2021 Jul 6;7:50. doi: 10.1038/s41421-021-00276-z (PMC8260710; doi:10.1038/s41421-021-00276-z)
Supplement: Supplementary file 1 — supplymentary data [file 41421_2021_276_MOESM1_ESM.docx]

**SUPPLEMENTARY INFORMATION**

**Materials and Methods**

**Ethics statement**

New Zealand white rabbits were obtained from the Laboratory Animal Center of Jilin University (Changchun, China). All animal studies were conducted according to experimental practices and standards approved by the Animal Welfare and Research Ethics Committee at Jilin University (SY202102005).

**Plasmid construction**

pCMV-PE2 was obtained from Addgene (#132775). The pegRNA, Spacer, sgRNA, pegRNA 3’ extension and nick sgRNA oligos were synthesized and clone in pBlyescriptSKII+ U6-sgRNA (F+E) empty vector (Addgene #74707) and pUC57-T7 vector (Addgene #51306) according to the previous study^1^.

**Cell culture and transfection**

The human kidney epithelial cells (HEK293FT) were cultured and transfected according to the previous study^2^. A total of 2000 ng PE2, 700 ng pegRNAs and 300 ng corresponding nick sgRNA were transfected into cells per 6-well. The sequencing of pegRNAs and sgRNAs are listed in Supplementary Table S1. The transfected cells were collected and performed to genomic DNA isolation (TIANGEN, Beijing, China) and PCR-amplified according to the manufacturer's instructions. Primers were listed in Supplementary Table S4.

The online software EditR (https://moriaritylab.shinyapps.io/editr_v10/)^3^ and TIDE/web tool (https://tide.deskgen.com/)^4^ were applied to estimate editing frequency using Sanger sequencing.

**mRNA and gRNA preparation**

All plasmids were linearized with *NotI* and transcribed *in vitro* using the HiScribe™ T7 ARCA mRNA kit (NEB). mRNA was purified using the RNeasy Mini Kit (Qiagen) according to the manufacturer’s protocol. The PegRNA and sgRNAs were amplified and transcribed *in vitro* using the MAXIscript T7 kit (Ambion) and purified using the miRNeasy Mini Kit (Qiagen) according to the manufacturer’s protocol.

**Zygote microinjection and embryo transfer**

The protocol used for the zygotes microinjection and embryo transfer has been described in detail in our previously published study^5^. Briefly, a mixture of PE2 mRNA (200ng/ul), sgRNA (50ng/ul) and nick sgRNA(50ng/ul) was co-injected into the cytoplasm of pronuclear-stage zygotes.

**Single-embryo PCR amplification and rabbit genotyping**

The single-embryo PCR amplification and rabbit genotyping were according to our previously study^5^. All the primers for genotyping are listed in Supplementary Table S4.

**Off-target assay**

Ten potential off-target sites (POTs) for each PegRNA and seven POTs for nick sgRNA were predicted to analyse site-specific edits according to Cas-OFFinder (http://www.rgenome.net/cas-offinder/)^6^. All primers for off-target assay are listed in Supplementary Table S5.

Targeted sites were amplified from genomic DNA using Q5 polymerase (NEB). Mutations were detected using deep sequencing of HiTOM analysis^7^.

**Real-time quantitative PCR (qRT-PCR) and Western Blotting**

The real-time quantitative PCR (qRT-PCR) was according to our previously study^8^. Primers used for RT-PCR are listed in Supplementary Table S7. The relative gene expression normalized to the GAPDH was determined by 2^−ΔΔCT^ formula. All the data of gene expression were performed at least three times.

For Western blotting, the ear tissues from *HEXA* ins TATC and WT rabbits were homogenized in 150μL of lysis buffer. The protein concentrations were measured by the Braford method (Bio-Rad). Anti-HEXA polyclonal antibody (1:5000, proteintech) and anti-β-actin monoclonal antibody (1:2000, proteintech) were used in this study.

**X-ray radiography**

X-ray radiography (47KV, 8mAn, 70cm) scans of the whole body was taken using a YEMA Radiography System with a digital camera (Varian, USA) attached to X-ray radiographer (Rotanode, Toshiba, Japan).

**Histology analysis**

The gastrocnemius, tibialis anterior and brain were collected from *HEXA* ins TATC rabbits and WT rabbits (euthanized at 3 and 28 days of age). The tissues were fixed in 4% paraformaldehyde at 4°C, dehydrated in increasing concentrations of ethanol (70% for 6 h, 80% for 1 h, 96% for 1 h and 100% for 3 h), cleared in xylene and embedded in paraffin for histological examination. The 5μm sections were cut for H&E and Masson’s trichrome staining. The stained sections were imaged with a Nikon TS100 microscope.

**Statistical analysis**

All data are expressed as the mean ±SEM, with at least three individual determinations in all experiments. The data were analysed with t-tests using GraphPad prism software 8.0. A probability of *p*<0.05 was considered statistically significant. A probability of *p*<0.05 was considered statistically significant. **p* <0.05, ***p* <0.01, ****p* <0.001.

**Reference**

1 Liu, Z. *et al.* Efficient and precise base editing in rabbits using human APOBEC3A-nCas9 fusions. *Cell discovery* **5**, 31, (2019).

2 Liang, M. *et al.* AcrIIA5 Suppresses Base Editors and Reduces Their Off-Target Effects. *Cells* **9**, (2020).

3 Kluesner, M. G. *et al.* EditR: A Method to Quantify Base Editing from Sanger Sequencing. *The CRISPR journal* **1**, 239-250, (2018).

4 Brinkman, E. K., Chen, T., Amendola, M. & van Steensel, B. Easy quantitative assessment of genome editing by sequence trace decomposition. *Nucleic acids research* **42**, e168, (2014).

5 Song, Y. *et al.* Efficient dual sgRNA-directed large gene deletion in rabbit with CRISPR/Cas9 system. *Cellular and molecular life sciences : CMLS* **73**, 2959-2968, (2016).

6 Bae, S., Park, J. & Kim, J. S. Cas-OFFinder: a fast and versatile algorithm that searches for potential off-target sites of Cas9 RNA-guided endonucleases. *Bioinformatics (Oxford, England)* **30**, 1473-1475, (2014).

7 Liu, Q. *et al.* Hi-TOM: a platform for high-throughput tracking of mutations induced by CRISPR/Cas systems. *Science China. Life sciences* **62**, 1-7, (2019).

8 Sui, T. *et al.* A novel rabbit model of Duchenne muscular dystrophy generated by CRISPR/Cas9. *Disease models & mechanisms* **11**, (2018).


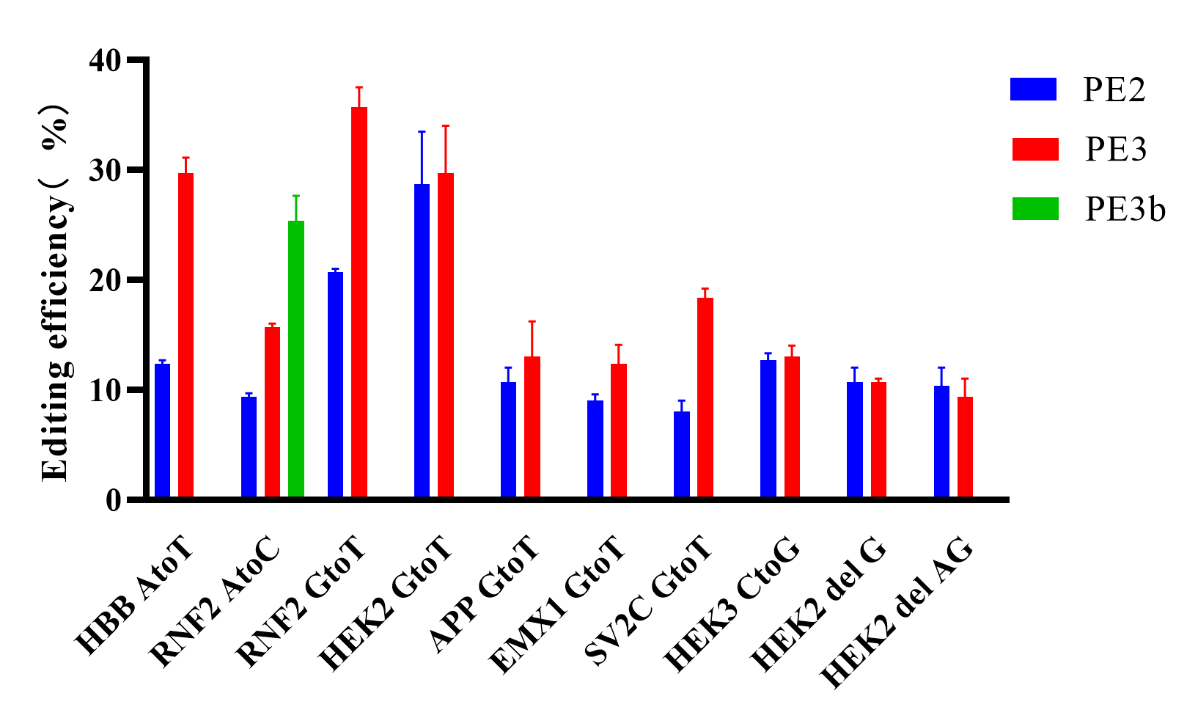


**Supplementary Fig. S1** The base substitutions and deletions efficiency of PE systems in HEK293FT cell.


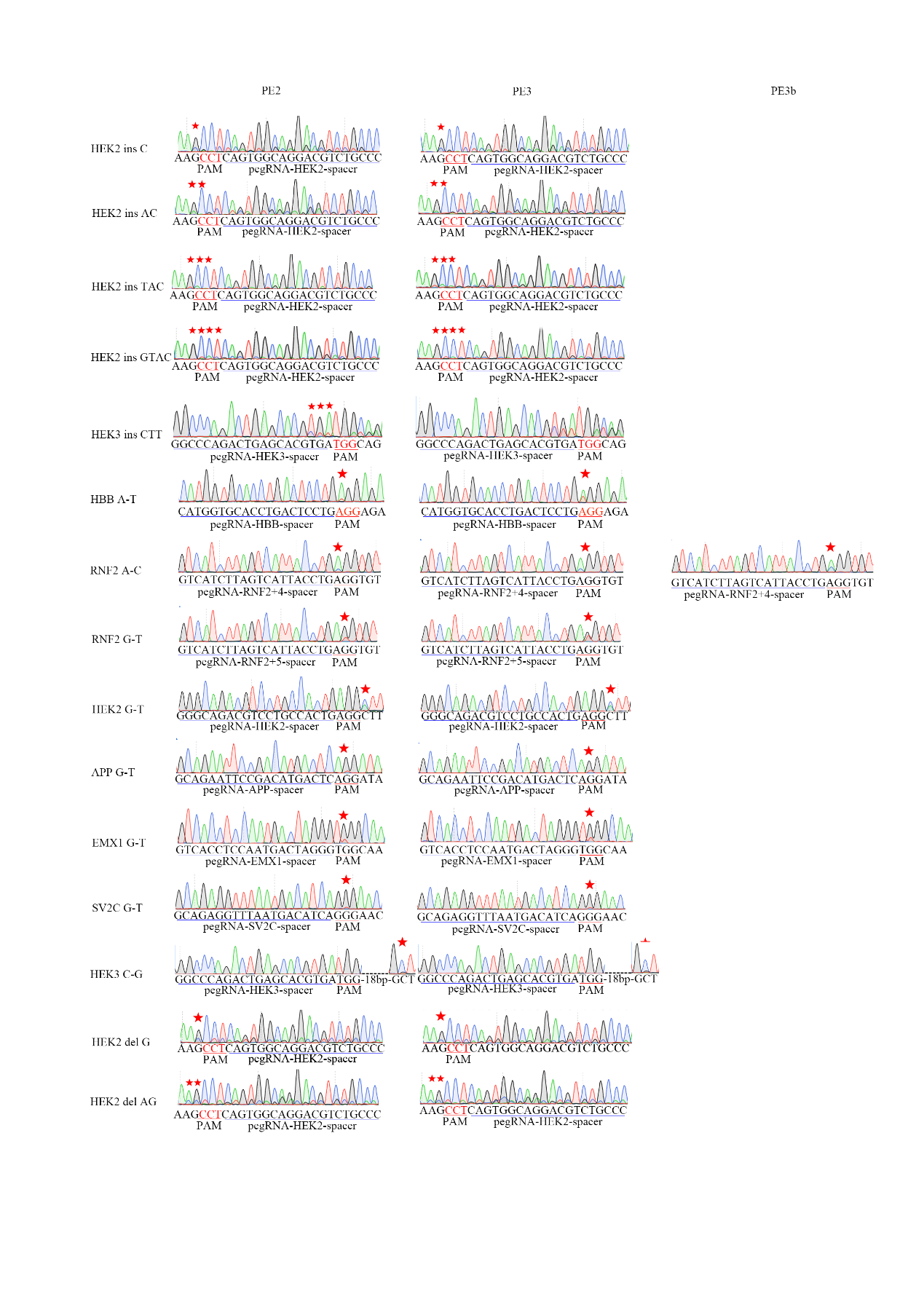


**Supplementary Fig. S2** Sanger sequencing chromatograms of PEs mediated base insertions conversions and deletions in human HEK293FT cells. The PAM sequence and spacer sequence of PegRNA are underlined in red and blue, respectively. Red stars indicate the targeted bases.


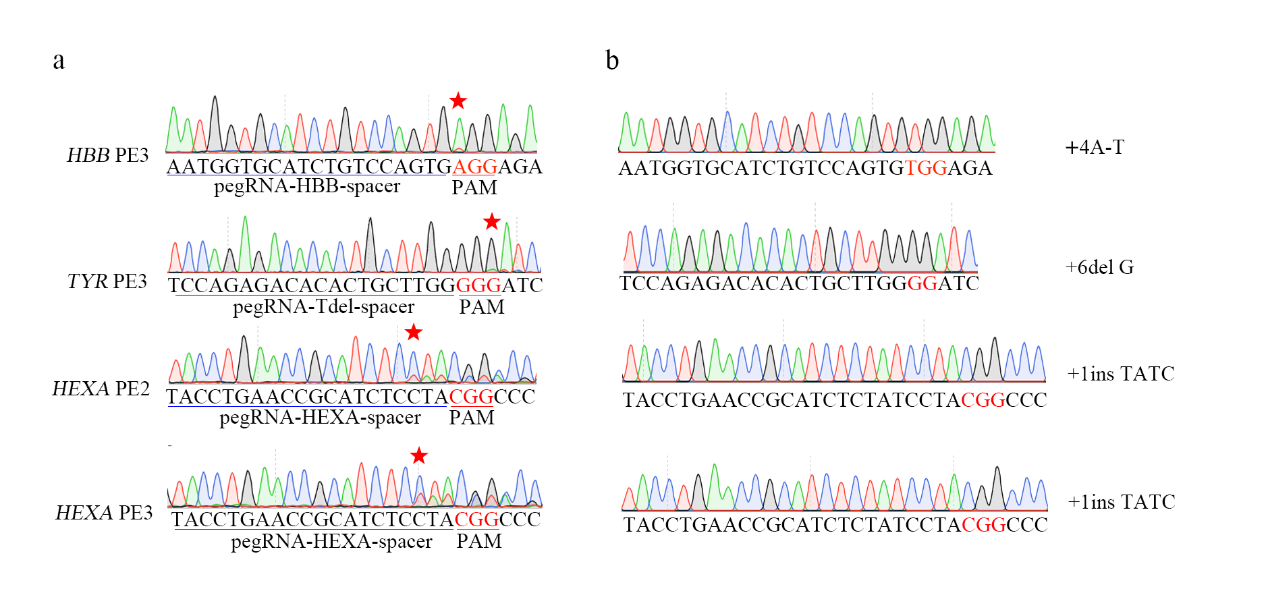


**Supplementary Fig. S3** Sanger sequencing (a) and T-cloning (b) chromatograms of PE mediated base transversion, deletion and insertion in rabbit embryos. The PAM sequence and spacer sequence of PegRNA are underlined in red and blue, respectively. Red stars indicate the targeted bases.

**
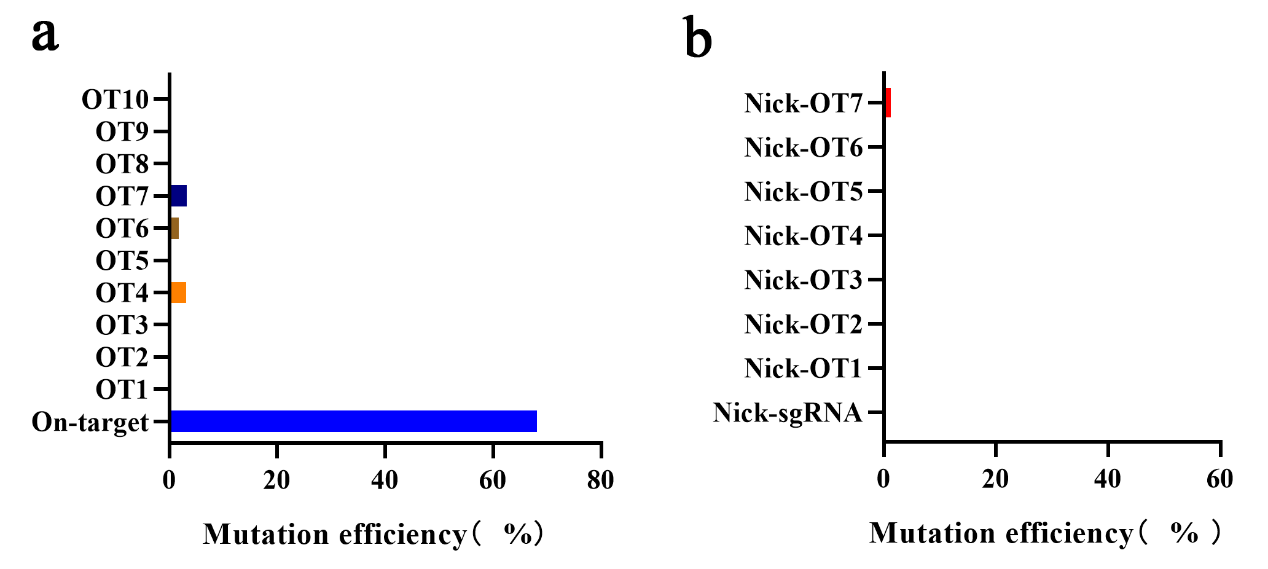
**

**Supplementary Fig. S4** Off-target analysis of *HEXA* ins TATC rabbit by deep sequencing of HiTOM analysis.

**a** The deep sequencing of 10 PegRNA potential off-target site in *HEXA* ins TATC rabbit.

**b** The deep sequencing of 7 nick sgRNA potential off-target site in *HEXA* ins TATC rabbit.


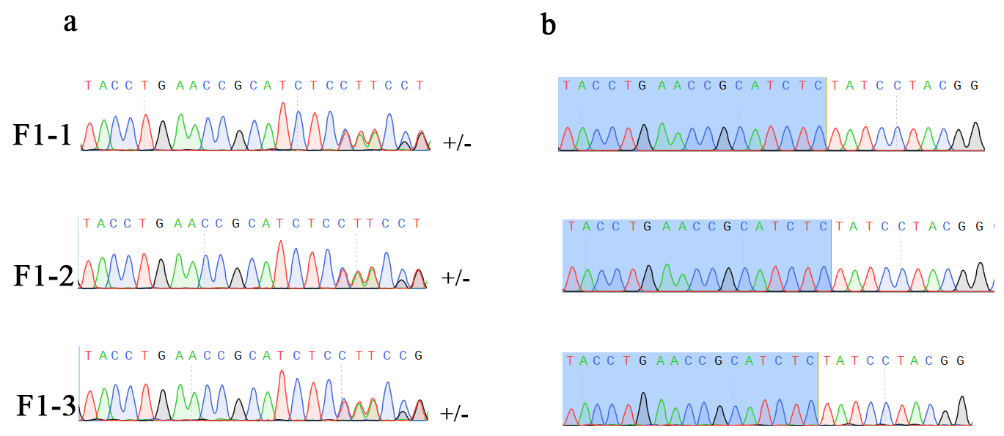


**Supplementary Fig. S5** Sanger sequencing (a) and T-cloning (b) chromatograms of *HEXA* ins TATC F1 rabbit pups.


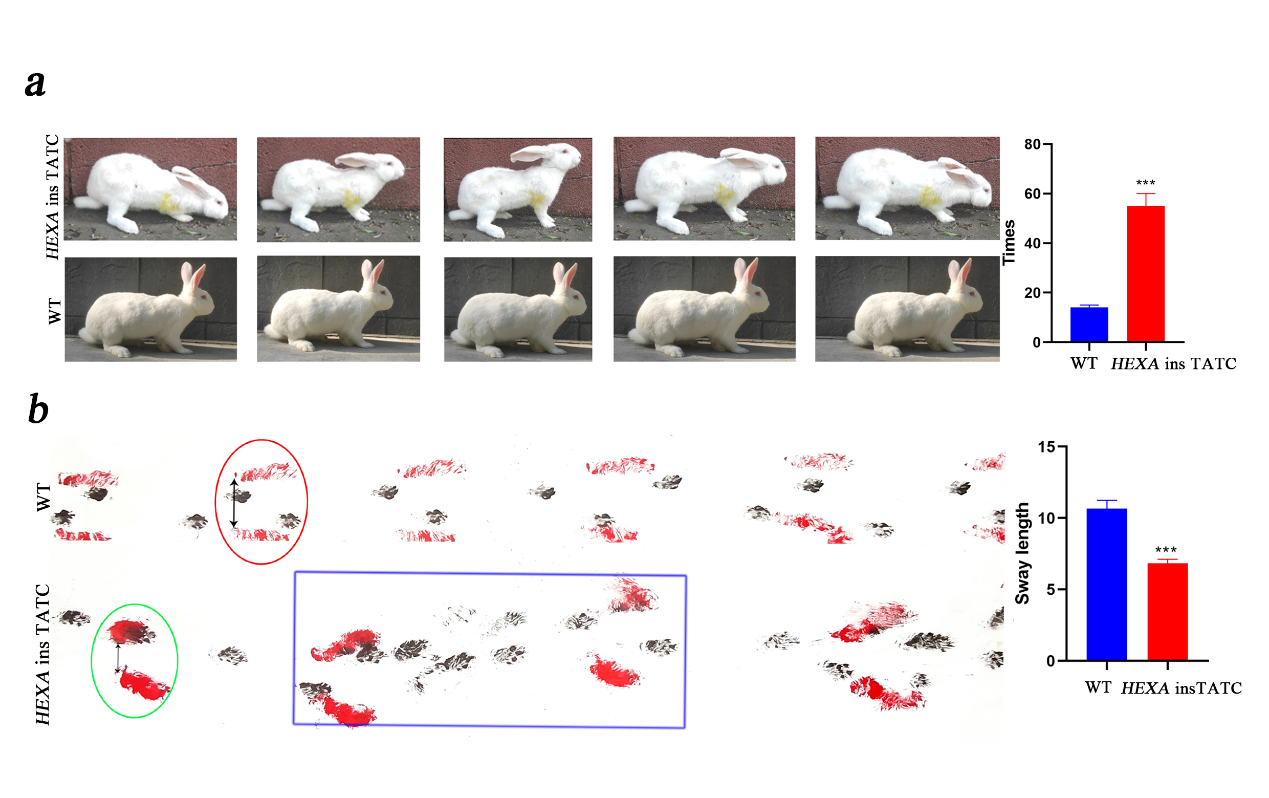


**Supplementary Fig. S6** The abnormal head raising and gait of *HEXA* ins TATC rabbit.

**a** The increased frequent of head raising in *HEXA* ins TATC rabbit.

**b** The decreased sway length of *HEXA* ins TATC rabbit. Red circle, the gait of WT rabbit; Green circle, the gait of *HEXA* ins TATC rabbit. Blue box, the abnormal movement of *HEXA* ins TATC rabbit.

**Supplementary Movie S1: Behaviour of *HEXA* ins TATC rabbit**

**Supplementary Movie S2: Behaviour of WT rabbit**

**Supplementary Table S1.** Sequences of PegRNA and sgRNA used in human HEK293FT cells.

| **pargana** | **Spacer sequence** | **3'extension sequence** | **PBS**  **length(nt)** | **RT template**  **length (nt)** |
| --- | --- | --- | --- | --- |
| *HBB* +4 AtoT | CATGGTGCACCTGACTCCTG | AGACTTCTCCACAGGAGTCAGGTGCAC | 13 | 14 |
| *RNF2* +4AtoC | GTCATCTTAGTCATTACCTG | AACGAACACCGCAGGTAATGACTAAGATG | 15 | 14 |
| *RNF2* +5GtoT | GTCATCTTAGTCATTACCTG | AACGAACACATCAGGTAATGACTAAGATG | 15 | 14 |
| *APP* +5GtoT | GCAGAATTCCGACATGACTC | ACTTCATATCATGAGTCATGTCGGAATTCT | 15 | 15 |
| *EMX1* +5GtoT | GTCACCTCCAATGACTAGGG | TGTGGTTGCCAACCCTAGTCATTGGAGGTG | 15 | 15 |
| *SV2C* +5GtoT | GCAACAAGATGTTTTGCCAAC | AGGTCTTGGCAAGTTGGCAAAACATCTTGT | 15 | 15 |
| *HEK2*+7GtoT | GGGCAGACGTCCTGCCACT | AGCCCAATCCTCAGTGGCAGGACGTCT | 13 | 14 |
| *HEK3*+26CtoG | GGCCCAGACTGAGCACGTGA | TGGAGGAACCAGGGCTTCCTTTCCTCTG  CCATCACGTGCTCAGTCTG | 13 | 34 |
| *HEK2*+7delG | GGGCAGACGTCCTGCCACT | AGCCCAACCTCAGTGGCAGGACGTCT |  |  |
| *HEK2*+8delAG | GGGCAGACGTCCTGCCACT | AGCCCACCTCAGTGGCAGGACGTCT |  |  |
| *HEK2*+7insC | GGGCAGACGTCCTGCCACT | AGCCCAACGCCTCAGTGGCAGGACGTCT |  |  |
| *HEK2*+7insAC | GGGCAGACGTCCTGCCACT | AGCCCAAACGCCTCAGTGGCAGGACGTCT |  |  |
| *HEK2*+7insTAC | GGGCAGACGTCCTGCCACT | AGCCCAATACGCCTCAGTGGCAGGACGTCT |  |  |
| *HEK2*+7insTAC | GGGCAGACGTCCTGCCACT | AGCCCAAGTACGCCTCAGTGGCAGGACGTCT |  |  |
| *HEK3*+1insCCT | GGCCCAGACTGAGCACGTGA | TCTGCCATCAAAGCGTGCTCAGTCTG | 13 | 12 |
| **Nick sgRNA** | **Spacer sequence** |  |  |  |
| *HBB* _-_+72 | CCTTGATACCAACCTGCCCA |  |  |  |
| *RNF2* _-_+41 | ATGTTTTGCTTAATGGTTGA |  |  |  |
| R*NF2* _-_+5 | GCGGTGTTCGTTGTAACTCA |  |  |  |
| *APP*_-_+49 | TTTGGCAAGACAAACAGTAG |  |  |  |
| *EMX1*_-_+49 | AGTCCAGCTTGGGCCCACGC |  |  |  |
| *SV2C*_-_+67 | AGGGAGTTGCTCATTGCCTC |  |  |  |
| *HEK2*_-_+44 | TGCCATTCTACCAACAATAG |  |  |  |
| *HEK3*_-_+57 | GCACATACTAGCCCCTGTCT |  |  |  |

**Supplementary Table S2.** Sequences of PegRNA and sgRNA used in rabbit embryos.

| **pegRNA** | **Spacer sequence** | **3'extension sequence** | **PBS**  **length(nt)** | **RT template**  **length (nt)** |
| --- | --- | --- | --- | --- |
| *HBB* AtoT | AATGGTGCATCTGTCCAGTG | AGACTTCTCCACACTGGACAGATGCAC | 13 | 14 |
| *TYR* del G | TCCAGAGACACACTGCTTGG | TTCGGATCCCCAAGCAGTGTGTCTC | 13 | 12 |
| *HEXA* ins TATC | TACCTGAACCGCATCTCCTA | AGTCAGGGCCGTAGGATAGAGATGCGGTTC | 12 | 14 |
| *HEXA* PBS-8 | TACCTGAACCGCATCTCCTA | AGTCAGGGCCGTAGGATAGAGATGCG | 8 | 14 |
| *HEXA* PBS-10 | TACCTGAACCGCATCTCCTA | AGTCAGGGCCGTAGGATAGAGATGCGGT | 10 | 14 |
| *HEXA* PBS-14 | TACCTGAACCGCATCTCCTA | AGTCAGGGCCGTAGGATAGAGATGCGGTTCAG | 14 | 14 |
| *HEXA* PBS-16 | TACCTGAACCGCATCTCCTA | AGTCAGGGCCGTAGGATAGAGATGCGGTTCAGGT | 16 | 14 |
| *HEXA* RT-10 | TACCTGAACCGCATCTCCTA | AGGGCCGTAGGATAGAGATGCGGTTC | 12 | 10 |
| *HEXA* RT-12 | TACCTGAACCGCATCTCCTA | TCAGGGCCGTAGGATAGAGATGCGGTTC | 12 | 12 |
| *HEXA* RT-16 | TACCTGAACCGCATCTCCTA | CCAGTCAGGGCCGTAGGATAGAGATGCGGTTC | 12 | 16 |
| *HEXA* RT-18 | TACCTGAACCGCATCTCCTA | TTCCAGTCAGGGCCGTAGGATAGAGATGCGGTTC | 12 | 18 |
| **Nick sgRNA** | **Spacer sequence** |  |  |  |
| *HBB* _-_+72 | AAAGGATACCAACCTGCCCA |  |  |  |
| *HEXA* _-_+44 | CGCTTCACCCTGAAATGCCA |  |  |  |
| *TYR* del _-_+52 | CTGTGCCAAGGCAGGAAACC |  |  |  |

**Supplementary Table S3.** The PegRNA (*HEXA* ins TATC) screen of RT template length (10-18) and PBS (8-16) in rabbit embryos.

| **Description** | **No.of zygetos** | **No.of blastocysts (%)** | **No. of embryos**  **With desired edits** | **Prime editing**  **Frequency (%)** |
| --- | --- | --- | --- | --- |
| RT-10 | 15 | 12(80) | 1 | 1.7 |
| RT-12 | 17 | 15(88) | 1 | 2.2 |
| RT-14 | 24 | 18(75) | 4 | 8-37.5 |
| RT-16 | 14 | 11(78.5) | 2 | 2.8-3.9 |
| RT-18 | 12 | 10(83) | 3 | 2.1-13.7 |
| PBS-8 | 15 | 11(73.3) | 2 | 1.7-2.9 |
| PBS-10 | 13 | 10(76.9) | 3 | 3.2-4.3 |
| PBS-12 | 24 | 18(75) | 4 | 8-37.5 |
| PBS-14 | 17 | 12(70.5) | 6 | 7.4-11 |
| PBS-16 | 14 | 10(71) | 5 | 13.7-23.1 |
| Cas9 HDR | 17 | 12(70.5) | 3 | 5.6-41.4 |

**Supplementary Table S4.** Primers used for DNA genotyping in 293FT cells and rabbit.

| **Description** | **Forward** | **Reverse** |
| --- | --- | --- |
| *HBB* | GCTGTCATCACTTAGACCTCAC | CTCCACATGCCCAGTTTCTAT |
| *RNF2* | TCTTTATTTCCAGCAATGTCTCAGG | AAGTCCATGGTTGGTGCTACA |
| *APP* | GGGTAGGCTTTGTCTTACAGTGTTAT | GGATGAACCAGAGTTAATAGGTCATT |
| *EMX1* | GGCCTCCTGAGTTTCTCATCT | TGTCCCTCTGTCAATGGCG |
| *SV2C* | TGGGGTTCTCCAGCTAACCA | TTCAGTGCAGGGAAAGCTAAGA |
| *HEK2* | CACAGGCTACCCCCTAAGTC | CCCTCAGCATTCAGCCACTAA |
| *HEK3* | GGGAAACGCCCATGCAATTA | GGTGCCCTGAGATCTTTTCCT |
| *HBB* | AGTTCAGGACTTGGGCATAAA | CAGGCGACTACTCTGTTTCTAC |
| *HEXA* | CGGCCAGACACCATCATC | TTTGTGCTGTCCACGTACTC |
| *TYR* | GCGACTCTTGGTGAGGAAA | AAAGATGCTGGGCTGAGTAG |

**Supplementary Table S5.** Primers used for off-target assay in rabbit.

| **Description** | **Forward** | **Reverse** |
| --- | --- | --- |
| Peg-OT1 | GTTGCGGAAATCCTGCTTCC | CAGGGGACTGTTGTCCGTTT |
| Peg-OT2 | ATGGGAAACCAGACAGACGG | AGTAACTGGGGGCGGTTTTC |
| Peg-OT3 | GCGGGGAGTAGTTCTGCTTT | TGAGCTGCTCTGCCTTTCAA |
| Peg-OT4 | AGGAGTGCCATTGTCTGTGG | AACCAGACCACCAGTAAAATTTGTT |
| Peg-OT5 | TAAAAACGTCAGTGCTGGGC | CTCTTTGCAATTAGGCCCCCT |
| Peg-OT6 | CTGGACAGCCACACCACAAA | GTGCTGGTGGTGCTAGTGATT |
| Peg-OT7 | ACAGTCATGCCCAATGAGGG | CTGTGCTGAACTGAGGAGCA |
| Peg-OT8 | CAAGGTTAAGGTGCAGGGACA | CTGAGCACAAAGGCTACTGC |
| Peg-OT9 | TGCCCACAAAGGAAAAGAGTCA | AGCCGTTTCCCAGAATGGAC |
| Peg-OT10 | TGTGCCCATTTCCAGGTTCC | AACGGTTCCAGGACAGACCA |
| Nick-sgRNA | CGTACTCTCCCCACATGCAG | GTGCTCTCGTGACGTGCT |
| Nick-OT1 | AGCACTGTTTGCTCCATGAC | AACATAGACCAGGTGAGAGCAT |
| Nick-OT2 | CAGGGCAATCCTCAAGGTGT | AGCTTGTGGCATAATGAAAAGGT |
| Nick-OT3 | CTGTTTTTCTTTCCCCTGGGC | CTGAGCCAGCAAGACGGAAG |
| Nick-OT4 | TGGACGAAAGGTGCGAAGG | GAACCTTCCTGAGAAGCGGC |
| Nick-OT5 | TCAGTCCACCTGCAAAAGGG | TACACATTGCTCTGCGACGA |
| Nick-OT6 | TGAGACGCCATCTCCACTTTC | CAGCGTTCCACTCACCTGG |
| Nick-OT7 | CTCCTGGAGGGCTACTTTGTG | ACCTTTGGAAGGGTCACATGG |

**Supplementary Table S6.** Primers used for mRNA in vitro transcription.

| **Description** | **Sequence** |
| --- | --- |
| IVT-T7-F | TAATACGACTCACTATAGG |
| IVT-HBB-R | AAAAGTGCATCTGTCCAGTGTGGAGAAGTCTGCAC |
| IVT-Tdel-R | AAAAGAGACACACTGCTTGGGGATCCGAAGCACCGACTCG |
| IVT-HEXA-R | GAACCGCATCTCTATCCTACGGC |
| IVT-HEXA-PBS-8R | CGCATCTCTATCCTACGGCCCTGACTGCAC |
| IVT-HEXA-PBS-10R | GAACCGCATCTCTATCCTACGGCCCTGACT |
| IVT-HEXA-PBS-14R | CTGAACCGCATCTCTATCCTACGGCCCTGA |
| IVT-HEXA-PBS-16R | ACCTGAACCGCATCTCTATCCTACGGCCCT |

**Supplementary Table S7.** RT-qPCR primers used for *HEXA* gene expression in rabbits.

| **Description (RT-PCR)** | **Forward** | **Reverse** |
| --- | --- | --- |
| *HEXA* | CCCTGGTACCTGAACCG | GATCACCAGGGCCTTCT |
| *GAPDH* | ATCCATTCATTGACCTCCACTAC | GTACTGGGCACCAGCATCAC |

**Supplementary Table S8.** The sgRNA and donor sequence used for HDR mediated HEXA ins TATC.

| **Description** | **Sequence** |
| --- | --- |
| sgRNA | TACCTGAACCGCATCTCCTA |
| donor | CCAACGCCAGCTTCCGGGCCCTGCTCTCCGCCCCCTGGTACCTGAACCGCATCTCTATCCTACGGCCCTGACTGGAAGAACTTCTACACAGTGGAGCCCCTGGCATTTCAGGGTGAAGCG |
